# Catalytic Performances of Cu/MCM-22 Zeolites with Different Cu Loadings in NH_3_-SCR

**DOI:** 10.3390/nano10112170

**Published:** 2020-10-30

**Authors:** Jialing Chen, Gang Peng, Tingyu Liang, Wenbo Zhang, Wei Zheng, Haoran Zhao, Li Guo, Xiaoqin Wu

**Affiliations:** 1 Key Laboratory of Hubei Province for Coal Conversion and New Carbon Materials, School of Chemistry and Chemical Engineering, Wuhan University of Science and Technology, Wuhan 430081, China; penggang@btrchina.com (G.P.); wustzhangwenbo@163.com (W.Z.); zhengwei321@126.com (W.Z.); Zhaohr290370@163.com (H.Z.); 2 Key Laboratory for Green Chemical Process of Ministry of Education, and Hubei Key Laboratory of Novel Reactor & Green Chemical Technology, School of Chemical Engineering & Pharmacy, Wuhan Institute of Technology, Wuhan 430205, China; ltingyu2006@yahoo.com

**Keywords:** Cu/MCM-22, NH_3_-SCR, Cu loading, hydrothermal aging

## Abstract

The NH_3_-SCR activities and hydrothermal stabilities of five *x*Cu/MCM-22 zeolites with different Cu loadings (*x* = 2–10 wt%) prepared by incipient wetness impregnation method were systematically investigated. The physicochemical properties of *x*Cu/MCM-22 zeolites were analyzed by XRD, nitrogen physisorption, ICP-AES, SEM, NH_3_-TPD, UV-vis, H_2_-TPR and XPS experiments. The Cu species existing in *x*Cu/MCM-22 are mainly isolated Cu^2+^, CuO*_x_* and unreducible copper species. The concentrations of both isolated Cu^2+^ and CuO*_x_* species in *x*Cu/MCM-22 increase with Cu contents, but the increment of CuO*_x_* species is more distinct, especially in high Cu loadings (>4 wt%). NH_3_-SCR experimental results demonstrated that the activity of *x*Cu/MCM-22 is sensitive to Cu content at low Cu loadings (≤4 wt%). When the Cu loading exceeds 4 wt%, the NH_3_-SCR activity of *x*Cu/MCM-22 is irrelevant to Cu content due to the severe pore blockage effects caused by aggregated CuO*_x_* species. Among the five *x*Cu/MCM-22 zeolites, 4Cu/MCM-22 with moderate Cu content has the best NH_3_-SCR performance, which displays higher than 80% NO*_x_* conversions in a wide temperature window (160–430 °C). Furthermore, the hydrothermal aging experiments (*x*Cu/MCM-22 was treated at 750 °C for 10 h under 10% water vapor atmosphere) illustrated that all the *x*Cu/MCM-22 zeolites exhibit high hydrothermal stability in NH_3_-SCR reactions.

## 1. Introduction

The burning of fossil fuels and intense human activities have caused sharp increases in the emissions of CO_2_, SO*_x_* (SO_2_ and SO_3_) and nitrogen oxides (NO*_x_*, mainly NO and NO_2_) in the atmosphere, which have resulted in a series of environmental problems, such as acid rain, photochemical smog, the greenhouse effect and haze [1,2,3]. As a very stable gas in the atmosphere with 114 years of half-life, NO*_x_* has attracted considerable attention in recent years due to its much higher greenhouse gas effect: 298 CO_2_ global warning equivalents in 100 years [4]. Selective catalytic reduction of NO*_x_* by ammonia (NH_3_-SCR) is one of the most widely used methods to reduce NO*_x_* emissions from both industrial off-gases and diesel engine exhausts due to its high efficiency and low cost, as it can use liquid ammonia or urea as the NH_3_ source [5,6].

Limited by the narrow temperature window (320–450 °C), insufficient hydrothermal stability and biotoxicity, conventional vanadia catalysts have gradually been replaced by nontoxic metal-based zeolite catalysts, especially Cu-based zeolites with a wide temperature window (200–500 °C) and high hydrothermal stability in NH_3_-SCR [4,5], owing to their unique properties such as high surface areas, considerable acidity and well-defined pore systems with stable structure which can sustain up to 800 °C [6,7]. In addition, due to the high redox activity of Cu species, Cu-based zeolites such as Cu/SSZ-13 [7,8], Cu/SAPO-34 [9,10], Cu/LTA [8,11] and Cu/SSZ-39 [12,13] have become the most used catalysts in NH_3_-SCR, especially in the low temperature range (200–350 °C) [13,14]. Currently, Cu/SSZ-13-based catalysts have been commercially applied in the treatment of diesel engine exhausts in the USA and Europe [5,14,15]. However, the high-temperature stability of Cu/SSZ-13 in NH_3_-SCR still cannot meet the industrial requirements [16]. According to the literature [17], the NH_3_-SCR activity of aluminum-rich (Si/Al < 8) Cu/SSZ-13 dramatically decreased after hydrothermal aging treatment at 750 °C for 12 h in air flow containing 10% H_2_O. As the NH_3_-SCR reactions usually occur in hydrothermal conditions at 200–550 °C with H_2_O (as one of the products) in the reaction atmosphere, Cu/SSZ-13 with low hydrothermal stability would result in frequent replacement of catalysts in practical applications. Lately, Cu/SAPO-34 with an identical CHA (Chabazite) structure to Cu/SSZ-13 was repeatedly found to be more stable than Cu/SSZ-13 during high-temperature (>700 °C) hydrothermal aging [18], and correspondingly became a better long-term SCR stable catalyst. However, Cu/SAPO-34 was proven to lack durability at low temperatures (<100 °C) in the presence of moisture [19,20]. Due to the destructive effect of H_2_O on the SAPO-34 framework, H_2_O in air could destroy Cu/SAPO-34 zeolite even at room temperature [21], which would lead to the loss of activity for Cu/SAPO-34 in NH_3_-SCR. Therefore, it is of great significance to develop novel Cu-based zeolite catalysts with both high activity and good hydrothermal stability in a wide temperature window.

MCM-22 with MWW topology is a kind of layered zeolite which has three different types of pores: two-dimensional sinusoidal channels with elliptical 10-membered ring cross-sections (0.41 × 0.51 nm), cylindrical supercages (0.71 nm in diameter and 1.82 nm in height) that are accessible through 10-membered ring (0.40 × 0.55 nm) windows and pockets on the external surface (0.71 nm in diameter and 0.70 nm in height) [22,23]. In comparison with SSZ-13 which can only be synthesized within narrow Si/Al ratios (5–50) with costly organotemplates such as N,N,N-trimethyladamantammonium hydroxide (TMAdaOH) [24], MCM-22 zeolites are synthesized in a wider composition range (Si/Al ratios of 10–200) with much lower costs [23,25]. As a result, MCM-22 zeolites are used as catalysts for the liquid-phase alkylation of benzene to cumene and ethylbenzene and as a good cracking zeolite additive for the fluid catalytic cracking (FCC) process in the petrochemical industry [26,27]. 

Corma and co-workers [28] found that Cu/MCM-22 zeolites were active catalysts with relatively high hydrothermal stability in selective catalytic reduction of NO with propane. Rutkowska and co-workers [29] found that Cu/MCM-22 zeolites could reach about 75% NO conversion with >90% N_2_ selectivity at only 180 °C in NH_3_-SCR. In addition, Cu/MCM-22 zeolites could still maintain 100% NO conversion and >90% N_2_ selectivity at 250–450 °C after hydrothermal aging in air with H_2_O at 550 °C for 3 h, which indicated that they were potential catalysts in NH_3_-SCR with good hydrothermal stability. Palella and co-workers [30] compared the decomposition of NO and N_2_O over Cu/MCM-22 and Cu/ZSM-5 zeolites, and revealed that Cu/MCM-22 displayed higher hydrothermal stability than Cu/ZSM-5. Lately, our group [22] found that the one-pot synthesized Fe/MCM-22 zeolite showed excellent activity in a wide temperature range (200–500 °C) in NH_3_-SCR. The above research results concerning the MCM-22 zeolites proved that Cu-based MCM-22 zeolites should be potential catalysts in NH_3_-SCR. However, detailed investigations about Cu/MCM-22 zeolites in NH_3_-SCR are still lacking.

According to previous studies [31], the main active sites of Cu-based zeolites in NH_3_-SCR reactions are isolated Cu^2+^ species or its hydrated form [Cu(OH)]^+^. Highly aggregated CuO*_x_* species show much lower activity than isolated Cu^2+^ species in NH_3_-SCR; besides, they will inevitably catalyze the ammonia oxidation side reactions at high temperatures, which is unfavorable for NH_3_-SCR. The chemical environment and distribution of active Cu species in Cu-based zeolites are often related to their Si/Al ratios, as the cationic Cu species need to electrostatically balance the negative charge generated by the AlO_4_^−^ tetrahedral structure in the zeolites [32,33,34,35]. When the Si/Al ratio of a zeolite is fixed, there should be an optimal Cu loading in Cu/zeolite catalysts, which can not only ensure the formation of enough active Cu species to guarantee high NH_3_-SCR activity, but also stabilize the zeolite framework, thereby improving the hydrothermal stability of catalysts [36,37,38]. For example, the first generation of commercial Cu/SSZ-13 catalyst developed by BASF company had a Si/Al ratio of 17.5 and an optimal Cu content of 2.8 wt% with 100% Cu^2+^ ion-exchange degree [16]. Further, the Si/Al of Cu/SSZ-13 catalyst was optimized to about 10 to guarantee enough acid sites in catalysts, as acid sites are also important for the NH_3_-SCR process; besides, the ion exchange degree of Cu^2+^ on Cu/SSZ-13 was adjusted to about 60% (Cu content is 2.8 wt%) to obtain the best catalytic activity and hydrothermal stability in NH_3_-SCR [31,39].

Therefore, in this work, a series of *x*Cu/MCM-22 zeolites with Si/Al ratios of about 15 (to ensure enough acid sites) and different Cu loadings (2–10 wt%) were prepared by incipient wetness impregnation method in order to investigate their catalytic activities and hydrothermal stabilities in NH_3_-SCR. The physicochemical properties of *x*Cu/MCM-22 zeolites were investigated by powder X-ray diffraction (XRD), N_2_ physical adsorption, NH_3_ temperature-programmed desorption (NH_3_-TPD), H_2_ temperature-programmed reduction (H_2_-TPR), inductively coupled plasma atomic emission spectroscopy (ICP-AES), X-ray photoelectron spectroscopy (XPS) and ultraviolet-visible diffuse reflectance spectra (UV-vis) experiments. The optimal Cu loading for H-MCM-22 with Si/Al ratio of 15 and the hydrothermal stability of *x*Cu/MCM-22 zeolites in NH_3_-SCR were clarified. The insights shown in this work should be of great benefit to the development of better NH_3_-SCR catalysts and the understanding of reaction processes.

## 2. Materials and Methods 

### 2.1. Catalyst Preparation

Parent H-MCM-22 zeolite (with Si/Al ratio of 15) was hydrothermally synthesized according to the previous procedures [22,23] by using sodium meta-aluminate (NaAlO_2_, 41 wt% Al_2_O_3_, 41 wt% Na_2_O, Sinopharm Chem. Reagent Co., Ltd. Shanghai, China), silica sol (40.5 wt% SiO_2_, Qingdao Haiyang Chem. Co., Ltd. Qingdao, China), hexamethyleneimine (HMI, 98 wt%, Aladdin industrial Co., Ltd. Shanghai, China), boric acid (99.8 wt%, Sinopharm Chem. Reagent Co., Ltd. Shanghai, China), sodium hydroxide (NaOH, 96 wt%, Sinopharm Chem. Reagent Co., Ltd. Shanghai, China) and deionized water. The synthesis gel had a molar composition of SiO_2_:0.033Al_2_O_3_:0.9H_3_BO_3_:1HMI:0.3NaOH:30H_2_O. The synthesis process was as follows: 0.73 g of NaOH, 0.80 g of NaAlO_2_ and 5.57 g of H_3_BO_3_ were dissolved in 45 g of deionized water. After stirring the mixture at room temperature for 10 min, 9.92 g of HMI was introduced under stirring. Finally, 14.81 g of silica sol was slowly added and it was further stirred for 3 h. The synthesis gel was then crystallized in a Teflon-lined stainless-steel autoclave at 170 °C for 5 days under rotation. The obtained products were filtered and washed to neutral. Then, the obtained products were dried overnight at 100 °C, and calcined at 560 °C for 10 h in air to remove the template molecules in the zeolites. H-MCM-22 (MCM-22 in hydrogen form) was prepared by ion-exchanging the calcined samples twice with NH_4_NO_3_ (99 wt%, Sinopharm Chem. Reagent Co., Ltd. Shanghai, China) aqueous solution (for example, 1 g zeolites were mixtures with 45 mL 1 mol/L NH_4_NO_3_ solution) at 80 °C for 5 h, which was then calcined in air at 550 °C for 6 h.

Cu-based MCM-22 zeolites were prepared by incipient wetness impregnation method. Typically, Cu (NO_3_)_2_·3H_2_O solution (0.38–1.89 g of Cu(NO_3_)_2_·3H_2_O (99–102%, Sinopharm Chem. Reagent Co., Ltd. Shanghai, China) dissolved in 23 mL deionized water) was mixed with 5 g of H-MCM-22 samples under constant stirring. The mixture solution was first treated at 40 KHz by ultrasound for 20 min and then stirred for 24 h at room temperature. After that, the products were dried at 100 °C for 12 h and then calcined in air at 550 °C for 6 h to obtain the *x*Cu/MCM-22 catalyst, where *x* represents the Cu loadings (*x* = 2, 4, 6, 8, 10 wt%, respectively). Hydrothermal aging treatment of *x*Cu/MCM-22 catalysts: portions of *x*Cu/MCM-22 zeolites were placed in a tube furnace, and then hydrothermally aged in air containing 10% water for 10 h at 750 °C; the obtained samples were denoted as *x*Cu/MCM-22-aged catalysts.

### 2.2. Catalyst Characterization

The X-ray powder diffraction (XRD) patterns collected on a PANalytical X’pert Pro MPD X-ray diffractometer (Philips, Eindhoven, Netherlands) was adjusted to a Cu *Kα* radiation of 154.06 pm in the range of 2*θ* from 3° to 50° with a scan speed of 4°/min. Using a paper by Xing and co-workers [40], the relative crystallinity (RC) of metal modified zeolites was obtained by dividing the sums of their diffraction peak intensities at 2θ of 14.3°, 22.7°, 23.7° and 26.0° by that of the reference sample (H-MCM-22 zeolite with a assumed 100% crystallinity).

The practical atomic composition of catalysts was measured by inductively coupled plasma atomic emission spectroscopy (ICP-AES, Autoscan16, TJA, Seymour Fisher Corporation, USA). 

Nitrogen adsorption/desorption isotherms were collected on a TriStar II 3020 gas adsorption analyzer (Micromeritics, Norcross, GA, USA). Before the measurement, zeolite samples were degassed for 8 h under high vacuum at 300 °C. The BET surface area (S*_BET_*) was calculated by Brunauer–Emmett–Teller (BET) method (focused on adsorption curve in the relative pressure range of 0.05 to 0.25). Total pore volume (V*_total_*) was determined at a nitrogen relative pressure of 0.99. The external surface area (S*_ext_*) and micropore volume (V*_micro_*) were calculated by t-Plot method. The micropore surface area (S*_micro_*) and mesopore volume (V*_meso_*) were calculated by the differences between S*_BET_* and S*_ext_*, and between V*_total_* and V*_micro_*, respectively.

With the assistance of field emission scanning electron microscope (Quanta 400 FEG, FEI Electron Optics, Hillsboro, OR, USA), scanning electron microscopy (SEM) images were taken to analyze the surface morphology of zeolite samples.

The ultraviolet-visible diffuse reflectance spectra (UV-vis) in 200–800 nm were collected on a Cary 5000 UV-vis-DRS spectrophotometer (Agilent Technologies Inc., Santa Clara, CA, USA, the diffuse reflectance was attached with a BaSO_4_ integrating sphere).

Prior to the H_2_ temperature-programmed reduction performed on a Chem Star chemisorption analyzer (Quantachrome Instrument Crop., Boynton Beach, FL, USA), 0.2 g of sample fixed in a quartz U-tube was pre-treated at 350 °C for 1 h and then cooled down to 50 °C (by a heating rate of 10 °C/min under Ar flow (30 mL/min)). Afterwards, in 10% H_2_/Ar (30 mL/min) flow, the temperature increased to 900 °C with a ramp of 10 °C/min. The signal fluctuation of H_2_ was recorded by using a thermal conductivity detector (TCD).

Before the temperature-programmed desorption of NH_3_ (NH_3_-TPD) experiment operated on a Chem Star chemisorption analyzer (Quantachrome Instrument Crop., Boynton Beach, FL, USA), 0.2 g catalysts were pre-treated with the same procedures as in H_2_-TPR experiments. Afterwards, samples were flushed with 10% NH_3_/He flow (30 mL/min) for 1 h at 50 °C, followed by pure He purging for 30 min to eliminate the gaseous/physically adsorbed NH_3_ species. Finally, catalysts were heated to 900 °C at a ramp of 10 °C/min in He flow (30 mL/min), during which the signal of NH_3_ was monitored by a TCD detector.

Aimed at investigating the surface properties and valence state, X-ray photoelectron spectroscopy (XPS) was collected on a Thermo ESCALAB 250 system (Thermo Fisher Scientific, Waltham, MA, USA) with Al Kα radiation (hν = 1486.6 eV). The C 1s (284.6 eV) was referenced so as to calibrate the binding energy.

### 2.3. Reaction Measurements

The catalytic performance of NH_3_-SCR was investigated in a fixed-bed flow reactor equipped with a quartz tube. Typically, 0.3 g 20–40 mesh catalyst was pre-treated in 5% O_2_/N_2_ flow (75 mL/min) at 120 °C for 30 min before increasing to 550 °C at a ramp of 10 °C /min. One hour later it was cooled down to 100 °C. Then the feed gas (300 mL/min) contained 500 ppm NO, 500 ppm NH_3_, 5 vol.% O_2_, and balanced N_2_ was introduced. The standard NH_3_-SCR was tested from 100 to 550 °C with 50 °C as a step. At each target temperature, a minimum maintenance time of 45 min was set to reach a steady state. Both the inlet and outlet concentrations of NO*_x_* (NO and NO_2_) were analyzed using a flue gas analyzer (KM950, Kane International Limited, Welwyn Garden City, UK). NO*_x_* conversion was calculated on the basis of:

(1) $${\mathrm{NO}}_{x}\;\mathrm{conversion}\;(\%)=\frac{C_{in,NO_{x}}-C_{out,NO_{x}}}{C_{in,NO_{x}}}\times 100\%$$

For NO oxidation experiments, the experimental steps are similar to the standard NH_3_-SCR experiments, while the feed gas was: 500 ppm NO, 5 vol.% O_2_ and balanced N_2_. The NO conversion was calculated on the basis of:

(2) $$\mathrm{NO}\;\mathrm{conversion}\;(\%)=\frac{C_{in,NO}-C_{out,NO}}{C_{in,NO}}\times 100\%$$

The subscripts “in” and “out” in Equations (1) and (2) represent the inlet and outlet concentrations of NO*_x_* or NO, respectively.

## 3. Results and Discussions

### 3.1. Textural and Structural Properties

**Figure 1:**
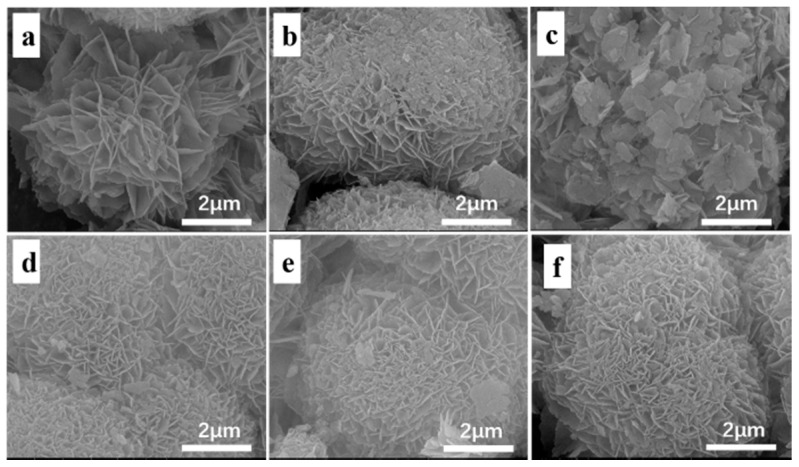
SEM images of H-MCM-22 (**a**) and *x*Cu/MCM-22 zeolites: (**b**) 2Cu/MCM-22, (**c**) 4Cu/MCM-22, (**d**) 6Cu/MCM-22, (**e**) 8Cu/MCM-22, (**f**) 10Cu/MCM-22.

**Figure 2:**
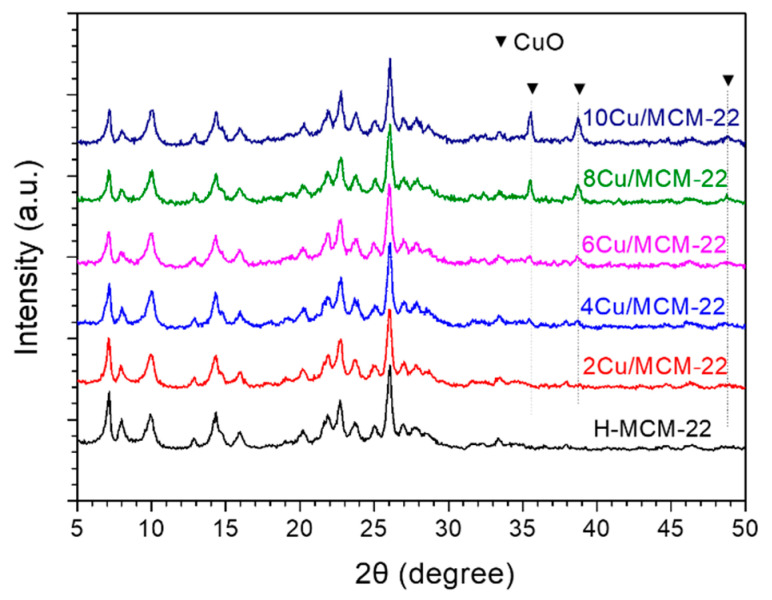
XRD patterns of H-MCM-22 and *x*Cu/MCM-22 zeolites.

Figure 1 shows the SEM images of H-MCM-22 (Figure 1a) and *x*Cu/MCM-22 zeolites (Figure 1b–f). All the zeolites had the disk-like shape of typical MWW zeolites with a similar particle size of around 1 µm in diameter and 50–100 nm in thickness, illustrating that the introduction of copper caused only minor changes in the textural properties of MCM-22 crystals. The XRD patterns of H-MCM-22 and *x*Cu/MCM-22 zeolites are shown in Figure 2, which exhibit typical diffraction peaks for MWW framework structures without impurities [23]. As shown in Table 1, the relative crystallinities (RC) of all the zeolites are similar and in the range of 93–102%, supporting that the introduction of copper into H-MCM-22 zeolites does not destroy the framework structure of MCM-22 zeolites.

In addition, as shown in Figure 2, small diffraction peaks at 2θ of 35.6°, 38.7° and 48.8° gradually emerge with the increasing of Cu contents, which indicates the formation of CuO nanoparticles in *x*Cu/MCM-22 zeolites [41,42]. As displayed in Table 1, the actual copper contents in *x*Cu/MCM-22 were close to their theoretical values (0, 2, 4, 6, 8, 10 wt%, respectively), illustrating that the Cu contents in *x*Cu/MCM-22 zeolites can be easily adjusted through incipient wetness impregnation method. As shown in Figure S1, a positive relationship between Cu content and the intensity of diffraction peak representing CuO was observed, indicating that the concentration of aggregated CuO nanoparticles gradually increased with Cu loadings in *x*Cu/MCM-22.

The quantitative results of nitrogen physisorption experiments are shown in Table 1 and Figure S2. As displayed in Table 1 and Figure S2, in comparison with parent H-MCM-22, both the BET surface area and total pore volume in 2Cu/MCM-22 decreased about 20%. When further increasing the Cu loading to 4 wt%, the loss percentage values in Figure S2B for 4Cu/MCM-22 only slightly changed. However, when Cu loading exceeded 4 wt%, the loss percentages of BET surface area and total pore volume for 6Cu/MCM-22, 8Cu/MCM-22 and 10Cu/MCM-2 dramatically decreased to 30–40%, which suggests that the formation of aggregated CuO species caused severe pore blockage in MCM-22 zeolites, supported by XRD results in Figure 2. Moreover, as shown in Table 1, the external surface area (S*_ext_*) and mesopore volume (V*_meso_*) only slightly changed from 2Cu/MCM-22 to 10Cu/MCM-22, which demonstrates that aggregated CuO*_x_* species mainly block the micropores in MCM-22 zeolites. Furthermore, comparing the loss of BET surface area (S*_BET_*) with micropore surface area (S*_micro_*) in Figure S2A, or the loss of total pore volume (V*_tota_*_l_) with micropore volume (V*_micro_*) in Figure S2B, it can be concluded that when Cu loading exceeds 6 wt%, the losses of S*_micro_* and V*_micro_* are much larger than those of S*_BET_* and V*_total_*, which indicates the severe blockage of microporous pore in zeolites at high Cu loadings.

In general, *x*Cu/MCM-22 zeolites with different Cu loadings have similar relative crystallinities, crystal size and surface morphology. However, aggravated CuO nanoparticles gradually formed with increasing Cu loadings, which led to the decrease of micropore surface area and micropore pore volume in *x*Cu/MCM-22. The pore blockage effect of CuO species in *x*Cu/MCM-22 was significant when the Cu loadings exceeded 4 wt%, which meant *x*Cu/MCM-22 lost 30–40% of BET surface area and total pore volume.

### 3.2. Acidity of Catalysts

A good NH_3_-SCR catalyst should have appropriate amounts of acid sites to promote ammonia adsorption and activation at the active sites during the reaction processes [36,43,44]. Thus, the temperature-programmed desorption of NH_3_ (NH_3_-TPD) was performed in order to investigate the acid properties of H-MCM-22 and *x*Cu/MCM-22 zeolites used in this work.

**Figure 3:**
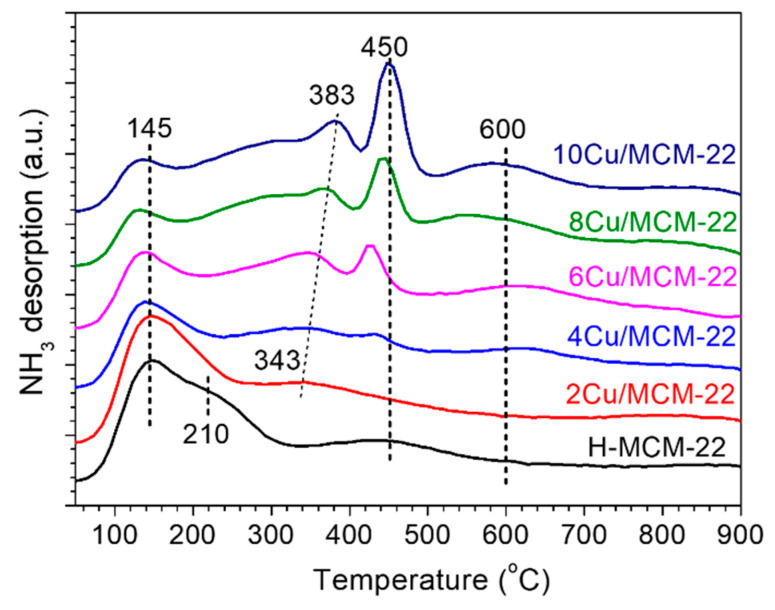
NH_3_-TPD profiles of H-MCM-22 and *x*Cu/MCM-22 zeolites.

As shown in Figure 3, three TPD peaks were observed at about 145, 210 and 450 °C in H-MCM-22 zeolite, which can be attributed to NH_3_ desorbed from the weak, medium and strong acid sites, respectively [45]. As all of the samples in Figure 3 have similar Si/Al ratios, the different acid densities of *x*Cu/MCM-22 zeolites can be mainly attributed to their different Cu loadings. In comparison with H-MCM-22, the TPD peak at 450 °C in 2Cu/MCM-22 dramatically decreased while the peak at 343 °C increased, which suggests that the introduced Cu species not only covered some of the strong acid sites but also formed some medium acid sites in 2Cu/MCM-22 [46]. When further increasing Cu loading to 10 wt% (from 2Cu/MCM-22 to 10Cu/MCM-22), the peak in 343 °C gradually changed to 383 °C, and two peaks at about 450 °C and 600 °C obviously emerged. As the intensities of peaks at 450 and 600 °C gradually increased with Cu loadings, we deduced that they may be related with Cu species.

According to the above speculations, the NH_3_-TPD profiles in Figure 3 were deconvoluted into three or six peaks, as shown in Figure S3. Correspondingly, the quantitative results of NH_3_-TPD experiments are summarized in Table 2. All the zeolites in Table 2 have similar total acid density (in the range of 1062 to 1276 µmol/g). However, the concentrations of weak, medium and strong acid sites in H-MCM-22 and *x*Cu/MCM-22 are apparently different. As the Cu loadings increased from 0 to 10 wt% (from H-MCM-22 to 10Cu/MCM-22), the densities of weak and medium acid sites gradually decreased from 255 µmol/g to 102 µmol/g, and from 446 µmol/g to 140 µmol/g, respectively. In contrast, the density of strong acid sites dramatically increased from 361 µmol/g to 1034 µmol/g. According to the literature [22,47,48,49,50], the results in Table 2 demonstrate that some of the weak and medium acid sites were covered by Cu species, but the introducing of Cu species also led to the increasing of strong acid sites. As shown in Figure 3, the shift of TPD peaks towards higher temperatures also confirms that the global acid strength of *x*Cu/MCM-22 zeolites gradually increases with Cu loadings, which is in agreement with the quantitative results in Table 2.

It should be noted that the parent H-MCM-22 zeolite contains boron elements because H_3_BO_3_ was used in the synthesis process of H-MCM-22 in order to increase the crystallinity and yield of H-MCM-22 zeolites. However, as boron can only form extremely weak acid sites, and can be easily removed in the following NH_4_NO_3_ ion-exchange procedures [22], it should have minor effects on the acidity of H-MCM-22 and *x*Cu/MCM-22 zeolites.

In general, the content of Cu in H-MCM-22 has little influence on the total acid density of *x*Cu/MCM-22 zeolites, but the global acid strength of *x*Cu/MCM-22 zeolites increases with Cu loadings, which may result in different NH_3_ adsorption and activation abilities of *x*Cu/MCM-22 zeolites in NH_3_-SCR.

### 3.3. Characterization of Cu Species

**Figure 4:**
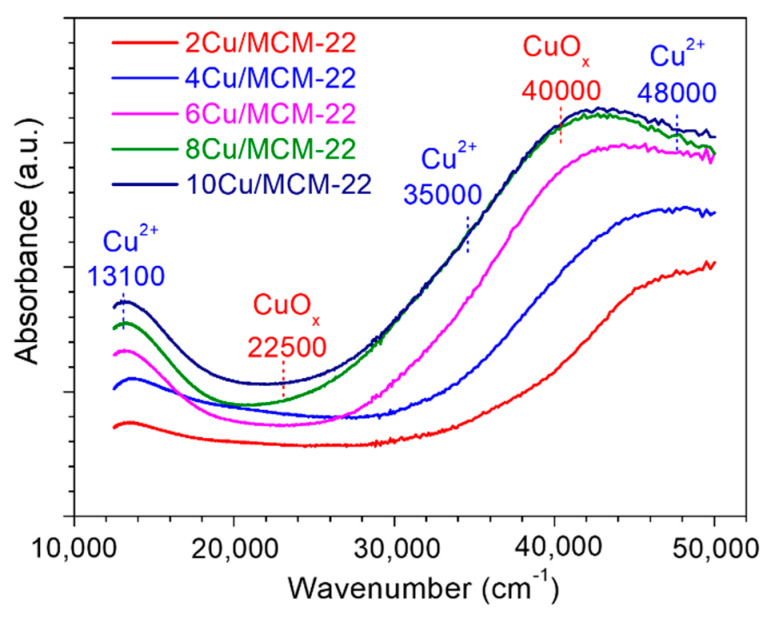
UV-vis spectra of *x*Cu/MCM-22 zeolites with different copper contents.

The existing states of Cu species in *x*Cu/MCM-22 zeolites were characterized by UV-vis spectroscopy, as shown in Figure 4. According to previous literature [51,52], the UV bands at around 13,100 cm^−1^ can be attributed to the d–d transitions of Cu^2+^ in a distorted octahedral configuration; the broad bands at around 35,000 and 48,000 cm^−1^ were caused by O^2-^→Cu^2+^ charge transfer transitions; all of those bands are characteristic of the isolated Cu^2+^ species. The UV bands near 22,500 and 40,000 cm^−1^ are assigned to the d–d transition and charge transfer transition of Cu with octahedral environment in CuO*_x_* species [42,53,54,55].

As shown in Figure 4, the UV band at around 48,000 cm^−1^ representing isolated Cu^2+^ species was observed for 2Cu/MCM-22 zeolite, while no bands at 22,500 and 40,000 cm^−1^ corresponding to CuO*_x_* species were observed, which suggests that most of the Cu species in 2Cu/MCM-22 were isolated Cu^2+^ species, in accordance with XRD results in Figure 2. With the increase of Cu loadings, the UV band at 40,000 cm^−1^ in 4Cu/MCM-22 emerged, indicating the formation of CuO*_x_* species. By further increasing Cu loadings (from 4Cu/MCM-22 to 10Cu/MCM-22), the UV absorption bands at 40,000 and 22,500 cm^−1^ rapidly increased; meanwhile, the band intensity at 40,000 cm^−1^ exceeds that at 48,000 cm^−1^. Those results illustrate that for *x*Cu/MCM-22 zeolites with high Cu loadings (6–10 wt%), most of the Cu species in the zeolites are CuO*_x_* species, though the concentrations of isolated Cu^2+^ species in *x*Cu/MCM-22 also increase with Cu loadings, as reflected by the increased intensity of the bands at 13,100 cm^−1^. As a whole, the UV-vis results reveal that both isolated Cu^2+^ and CuO*_x_* species in *x*Cu/MCM-22 increase with Cu loadings; besides, Cu species are mainly in the form of isolated Cu^2+^ species for 2Cu/MCM-22, while for *x*Cu/MCM-22 with high Cu loadings (>4 wt%), CuO*_x_* species become dominant.

**Figure 5:**
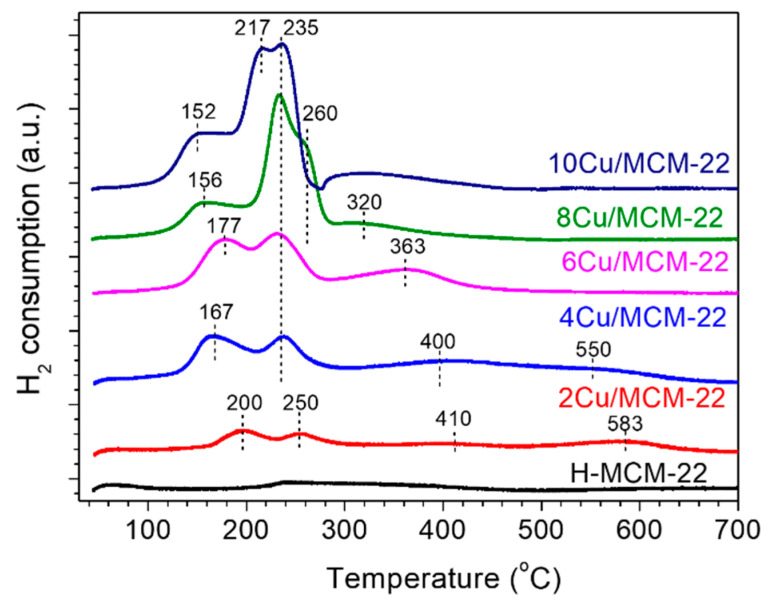
H_2_-TPR profiles of H-MCM-22 and *x*Cu/MCM-22 zeolites.

Temperature-programmed reduction with hydrogen (H_2_-TPR) experiments were conducted in order to further investigate the Cu species in *x*Cu/MCM-22 zeolites. As shown in Figure 5, no obvious reduction peak was observed for parent H-MCM-22 zeolite. In contrast, the reduction peaks of five *x*Cu/MCM-22 zeolites are complex. For example, four reduction peaks at about 200, 250, 410 and 583 °C can be distinguished in 2Cu/MCM-22, while for 8 Cu/MCM-22 and 10 Cu/MCM-22, only severely overlapped reduction peaks were observed. According to the literature [56,57], the peak at 250 °C in 2Cu/MCM-22 can be attributed to the reduction of CuO nanoparticles to Cu^0^, which indicates that highly dispersed CuO nanoparticles exist in 2Cu/MCM-22, even though they cannot be observed by XRD (Figure 2) due to the detection limitation. On the other hand, the reduction of isolated Cu^2+^ species in zeolites needs two steps due to the strong electrostatic interactions between isolated Cu^2+^ species and the zeolite framework [58]: the isolated Cu^2+^ first be reduced into Cu^+^ and then Cu^+^ be reduced into Cu^0^ at higher temperatures during H_2_-TPR experiments. Thus, the reduction peak at 200 °C in 2Cu/MCM-22 can be attributed to the reduction of isolated Cu^2+^ to Cu^+^; the peaks at 410 °C and 583 °C resulted from the reduction of Cu^+^ to Cu^0^ species [9,31,59]. The reason for the occurrence of two reduction peaks in the reduction of Cu^+^ to Cu^0^ may be due to multiple channel systems of MCM-22 zeolites, which form multiple ion-exchange sites for isolated Cu^2+^ species with significantly different reducibility. According to Wasowicz and co-workers [60], there are at least three different ion-exchange sites of Cu^2+^ ions in MCM-22 zeolite, among which the most favorable sites of Cu^2+^ ions are located near the five-ring presented in the intralayer of the two-dimensional sinusoidal channel system and in the interlayer large supercages.

With the increase of Cu loading, the reduction peaks of *x*Cu/MCM-22 zeolites in Figure 5 gradually shift to lower temperatures, though the total peak area increases. For 4Cu/MCM-22, four reduction peaks at around 167, 235, 400 and 550 °C can be distinguished, and only three peaks at 177, 235 and 363 °C were observed for 6Cu/MCM-22. As proven in previous studies [57,58], the reduction of Cu species becomes easier with the increase of nearest neighbor Cu species. Therefore, the reduction peak at about 177 °C in 6Cu/MCM-22 may be contributed to from both the reduction of CuO to Cu^0^ and of Cu^2+^ reduced to Cu^+^. By further increasing Cu loading to 10 wt%, the reduction peak of Cu^2+^ to Cu^+^ decreases to 152 °C; meanwhile, the peaks in the range of 200–300 °C dramatically increase and become severely overlapped. According to the literature [56,57], the overlapped peaks between 200 and 300 °C can be attributed to the reduction of CuO to Cu^0^ in different pore systems of MCM-22 zeolites, as their peak intensities greatly increase with Cu loadings. 

Based on the above assignments, the peak deconvolution results of H_2_-TPR experiments are displayed in Figure S4 and Table 3. Due to the complexity of Cu reduction behaviors in high Cu loadings, the results in Table 3 are just semi-quantitative because of the highly overlapped peaks, especially in 8Cu/MCM-22 and 10Cu/MCM-22. As shown in Figure 5, the reduction process I (Table 3) is easily overlapped with reduction process II, so we tentatively take the reduction process III as the representative of isolated Cu^2+^ species. As shown in Table 3, from 2Cu/MCM-2 to 10Cu/MCM-22, the relative peak area percentages of CuO increase from 14% to 58%, while the relative peak area percentages of isolated Cu^2+^ species decrease from 65% to 13%, both of which are consistent with the UV-vis results in Figure 4.

The quantitative H_2_ consumption results of Figure 5 are also presented in Table 3. The H_2_/Cu ratios of *x*Cu/MCM-22 zeolites were less than 1 (the theoretical H_2_/Cu ratio for the reduction process of Cu^2+^ to Cu^0^), which decreased from 0.73 to 0.60 with increasing Cu loadings. According to Yue and co-workers [61], the low H_2_/Cu ratios may be caused by the existence of unreducible copper species in *x*Cu/MCM-22 zeolites. As proven by Figure S5, no obvious peak can be observed for 6Cu/MCM-22, even when reduced at 900 °C during the H_2_-TPR experiment, which suggests that unreducible copper species indeed exist in *x*Cu/MCM-22, as the highly aggravated Cu species should be hardly accessible to H_2_ molecules and thus hard to be fully reduced.

In general, H_2_-TPR experiments illustrated that three kinds of Cu species—i.e., isolated Cu^2+^, CuO nanoparticles and unreducible copper species—exist in *x*Cu/MCM-22 zeolites. In addition, with the increment of Cu loadings, CuO nanoparticles gradually become the predominant Cu species in *x*Cu/MCM-22. Moreover, the reduction temperatures of *x*Cu/MCM-22 gradually shift to lower values with increasing Cu loadings, which may indicate the increase of reducibility for *x*Cu/MCM-22 in NH_3_-SCR.

**Figure 6:**
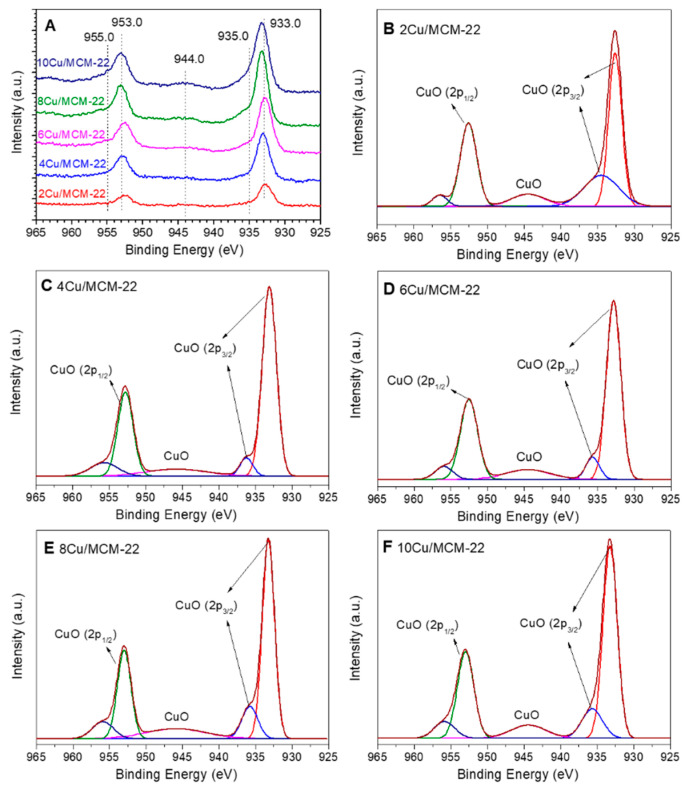
Cu 2p XPS spectra of *x*Cu/MCM-22 with different Cu contents: (**A**) original spectra and deconvolution spectra of (**B**) 2Cu/MCM-22, (**C**) 4Cu/MCM-22, (**D**) 6Cu/MCM-22, (**E**) 8Cu/MCM-22 and (**F**) 10Cu/MCM-22.

The content of surface Cu species and their chemical environment were further investigated by X-ray photoelectron spectroscopy (XPS). As presented in Figure 6A, at least five peaks can be distinguished in the Cu 2p XPS spectra of *x*Cu/MCM-22 zeolites. According to the literature [62,63,64,65], the Cu 2p_3/2_ peak at around 933 eV, the Cu 2p_1/2_ peak at around 953 eV and the 2p → 3d satellite peak at around 944 eV confirm the existence of CuO species in *x*Cu/MCM-22 zeolites, which is in accordance with the above XRD, UV-vis and H_2_-TPR results. Moreover, the asymmetry of the Cu 2p_3/2_ peak at about 933 eV can be deconvoluted into two contributions centered around 933 and 935 eV (as shown in Figure 6B–F), corresponding to dispersed CuO and CuO species which have electrostatic interactions with MCM-22 zeolite framework, respectively, according to previous reports [63,64,65]. Those results suggest that most of the Cu species existing on the surfaces of *x*Cu/MCM-22 zeolites are CuO nanoparticles, even for 2Cu/MCM-22 with the lowest Cu loading.

The quantitative XPS results of *x*Cu/MCM-22 are displayed in Table 4, in comparison with the ICP-AES results. As shown in Table 4, the Si/Al ratios of *x*Cu/MCM-22 derived from XPS are slightly lower than those from ICP-AES, suggesting that aluminum species are slightly rich on the surface of *x*Cu/MCM-22 zeolites. According to Zhu and co-workers [66], the enrichment of aluminum species on the surface of *x*Cu/MCM-22 after Cu introduction, especially in *x*Cu/MCM-22 with high Cu loadings (from 4Cu/MCM-22 to 10Cu/MCM-22), may be due to the formation of extra-framework Al species and migration of Al species to the surface of MCM-22 zeolites during the calcination process. On the contrary, the Si/Cu ratios of *x*Cu/MCM-22 obtained from XPS are apparently higher than those from ICP-AES, which indicates that most of the Cu species in *x*Cu/MCM-22 are concentrated in the bulk rather than on the surface. As shown in Table 1 and Figure S2B, the decreased BET surface areas and pore volumes of *x*Cu/MCM-22 with Cu loadings also support the enrichment of Cu species in the zeolite pore systems. In summary, Cu species on the surface of *x*Cu/MCM-22 mainly exist in the form of CuO, though most of the Cu species were concentrated in the bulk of *x*Cu/MCM-22 zeolites.

### 3.4. NH_3_-SCR Catalytic Performance

**Figure 7:**
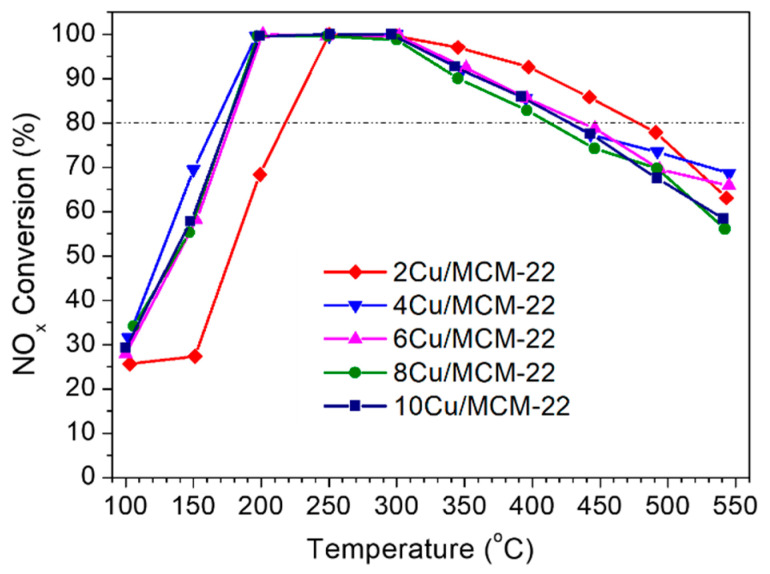
NO*_x_* conversion in NH_3_-SCR as a function of temperature over *x*Cu/MCM-22 zeolites.

As shown in Figure 7, the catalytic behaviors of five *x*Cu/MCM-22 zeolites are typical for NH_3_-SCR reactions. NO*_x_* conversions first increase with temperature, reaching the maximum conversions at 200–300 °C and then decrease with temperature after 300 °C, as the oxidation of ammonia with oxygen is highly favored at high temperatures [67].

Compared with 2Cu/MCM-22, the NO*_x_* conversions of 4Cu/MCM-22 at a low temperature range (<300 °C) increased, but at above 300 °C apparently decreased. In addition, the NO*_x_* conversion curves of 6Cu/MCM-22, 8Cu/MCM-22 and 10Cu/MCM-22 zeolites are almost overlapping, indicating their similar SCR activity in spite of difficult Cu loadings. Those results suggest that the NH_3_-SCR activity of *x*Cu/MCM-22 can be affected only at low Cu loadings (≤4 wt%); once Cu loading exceeds 4%, the catalytic activity has little association with Cu contents. In general, 4Cu/MCM-22 shows the best NH_3_-SCR activity among the five *x*Cu/MCM-22 zeolites, which exhibits higher than 80% NO*_x_* conversion in a wide temperature window (160–430 °C).

The active Cu species in NH_3_-SCR over Cu-based zeolites are quite complex, especially in the low temperature range (<300 °C) [14,15]. Gao and co-workers [32,37] found that [Cu(OH)]^+^ and dimer Cu^2+^ species were the main active centers in NH_3_-SCR below 300 °C due to the solvation effects between Cu^2+^ species and H_2_O at low temperatures. Besides, an ammonia inhibition effect was also observed on the rate-limiting copper re-oxidation step in NH_3_-SCR below 250 °C [68], which could suppress the oxidation of Cu^+^ to Cu^2+^ species and thus inhibit the NH_3_-SCR reactions. In contrast, the NH_3_-SCR reactions at high temperature (>300 °C) are less affected by the H_2_O solvation and ammonia inhibition effects; isolated Cu^2+^ species are widely accepted as the primary active sites [32,37]. However, the ammonia oxidation side reactions are highly favored at high temperatures, which are mainly catalyzed by aggregated CuO*_x_* species and can ultimately cause the decrease of NO*_x_* conversions.

As illustrated by UV-vis and H_2_-TPR experiments, concentrations of both of the isolated Cu^2+^ and CuO*_x_* species increased with Cu loadings. In addition, H_2_-TPR profiles in Figure 5 also reveal that the reducibility of *x*Cu/MCM-22 zeolites gradually increases with Cu loadings. Then, it is reasonable to expect that the NO*_x_* conversions of *x*Cu/MCM-22 in NH_3_-SCR below 300 °C should increase with Cu loadings. However, the results in Figure 7 contradict that assumption. According to the NH_3_-TPD results in Section 3.2, the acid strength of *x*Cu/MCM-22 zeolites increases with Cu loadings. As high acid strength may favor ammonia inhibition effect in low temperature NH_3_-SCR reactions [68], it can be suggested that the ammonia inhibition effect may be one of the causes for the decreased NO*_x_* conversions in *x*Cu/MCM-22 with increasing Cu loadings in NH_3_-SCR below 300 °C.

**Figure 8:**
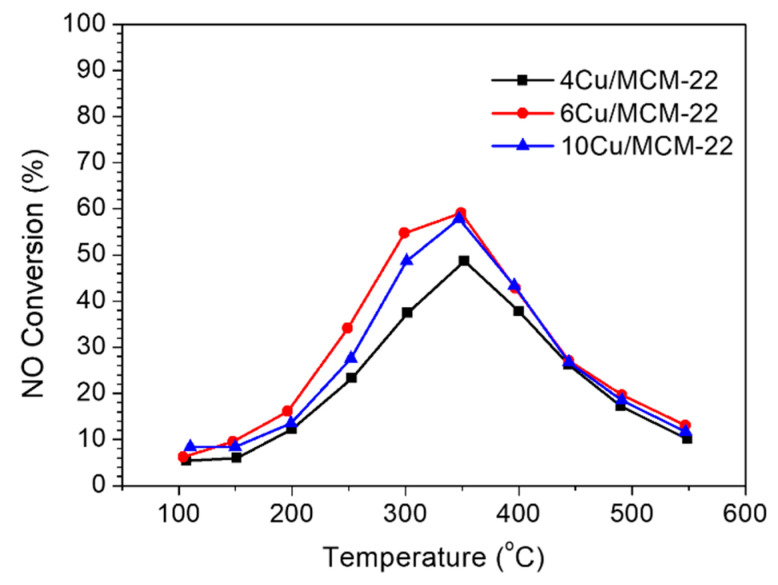
NO conversion in the NO oxidation reaction as a function of temperature over 4Cu/MCM-22, 6Cu/MCM-22 and 10Cu/MCM-22.

Moreover, as shown in Figure S2, due to the pore blockage effect, the BET surface areas and total pore volumes of *x*Cu/MCM-22 zeolites dramatically decrease when Cu loadings exceed 4 wt%, which may be another factor affecting the SCR activity of catalysts. In order to verify this speculation, NO oxidation experiments were conducted on 4Cu/MCM-22, 6Cu/MCM-22 and 10Cu/MCM-22. As shown in Figure 8, the NO conversions of all the catalysts first increased with temperature and then rolled over above 350 °C, due to thermodynamic limitations of NO oxidation reaction, in accordance with previous studies [69,70].

According to references [71,72,73], NO oxidation reactions are predominately catalyzed by dimer Cu^2+^ species and CuO*_x_* species; the isolated Cu^2+^ species have little NO oxidation activity. From XRD, UV-vis, H_2_-TPR and XPS results, the concentration of CuO*_x_* species in *x*Cu/MCM-22 significantly increases with Cu loadings, which should lead to increased NO oxidation activity. However, the NO conversions of three catalysts in Figure 8 are in the order of 6Cu/MCM-22 > 10Cu/MCM-22 > 4Cu/MCM-22. As shown in Figure S2 and Table 1, 10Cu/MCM-22 shows apparently lower total pore volume (0.34 cm^3^ g^−1^) and surface area (290 m^2^ g^−1^) than 4Cu/MCM-22 (0.40 cm^3^ g^−1^ and 404 m^2^ g^−1^) and 6Cu/MCM-22 (0.39 cm^3^ g^−1^ and 391 m^2^ g^−1^), which means that the pore blockage effect is more severe in 10Cu/MCM-22 than other two zeolites. Therefore, though more CuO*_x_* species exist in 10Cu/MCM-22, large amounts of aggravated CuO*_x_* nanoparticles severely block the pore systems of 10Cu/MCM-22 zeolites, which makes most of the active sites in 10Cu/CMM-22 inaccessible to reactant molecules and ultimately leads to decreased NO oxidation activity. As NO oxidation is one of the reactions occurring in NH_3_-SCR, it is reasonable to believe that the pore blockage effect also has a significant impact in NH_3_-SCR over *x*Cu/MCM-22 zeolites, especially when Cu loadings exceed 4 wt%.

Therefore, the complex NH_3_-SCR performances of *x*Cu/MCM-22 zeolites with different Cu loadings in Figure 7 may have resulted from a combination of multiple factors, such as Cu loadings, the ammonia inhibition effect and the pore blockage effect. At low Cu loadings (≤4 wt%), the NH_3_-SCR activity of *x*Cu/MCM-22 is mainly affected by the concentration of active isolated Cu^2+^ species and the ammonia inhibition effect. The increase of isolated Cu^2+^ species with Cu loadings in *x*Cu/MCM-22 will promote the NH_3_-SCR reactions. However, the increased acid strength with Cu loadings (as proven by NH_3_-TPD) may cause a severe ammonia inhibition effect, which will deteriorate the NH_3_-SCR reactions, especially in a low temperature range (<250 °C). When Cu loading exceeded 4 wt%, due to the formation of large amounts of aggregated CuO*_x_* species, the pore blockage effect became a dominant factor deciding the NH_3_-SCR performance of *x*Cu/MCM-22 zeolites, which resulted in the similar NH_3_-SCR performances of 6Cu/MCM-22, 8Cu/MCM-22 and 10Cu/MCM-22 in Figure 7. As a result, 4Cu/MCM-22 showed the best NH_3_-SCR activity among the five *x*Cu/MCM-22 zeolites by balancing different influencing factors.

### 3.5. Hydrothermal Stability of xCu/MCM-22 in NH_3_-SCR

**Figure 9:**
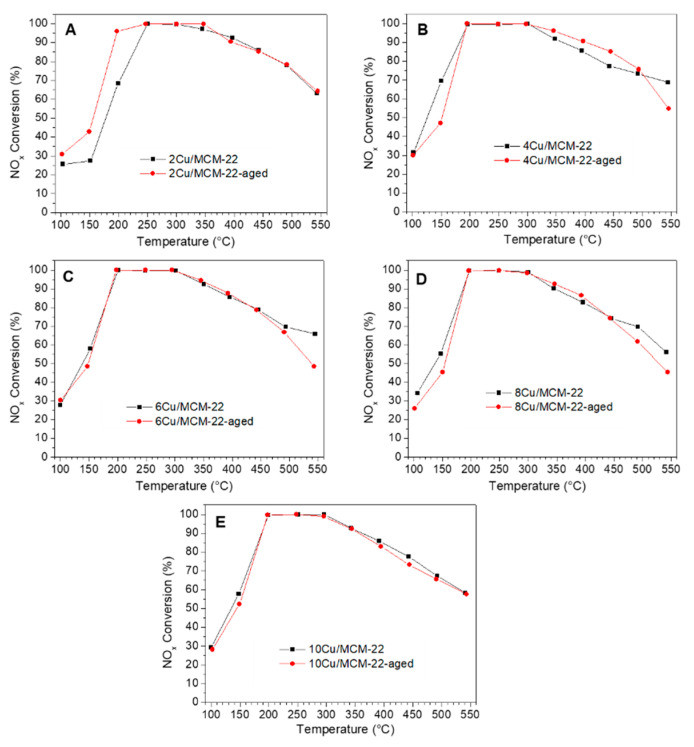
Comparison of NH_3_-SCR activity of *x*Cu/MCM-22 (before hydrothermal aging) and *x*Cu/MCM-22-aged (after hydrothermal aging) with different Cu contents: (**A**) 2Cu/MCM-22 and 2Cu/MCM-22-aged; (**B**) 4Cu/MCM-22 and 4Cu/MCM-22-aged; (**C**) 6Cu/MCM-22 and 6Cu/MCM-22-aged; (**D**) 8Cu/MCM-22 and 8Cu/MCM-22-aged; (**E**) 10Cu/MCM-22 and 10Cu/MCM-22-aged.

The NH_3_-SCR activities of *x*Cu/MCM-22 zeolites before and after hydrothermal aging are displayed in Figure 9. After hydrothermal aging in air containing 10% water at 750 °C for 10 h, the NO*_x_* conversions of *x*Cu/MCM-22 only slightly decreased, indicating that *x*Cu/MCM-22 zeolites have high hydrothermal stability in NH_3_-SCR.

In comparison with 2Cu/MCM-22, the NO*_x_* conversion of 2Cu/MCM-22-aged at 100–200 °C significantly increased, as shown in Figure 9A. According to the literature [74,75,76,77], some of CuO*_x_* species (with low activity in NH_3_-SCR) on the surface of Cu-SAPO-34 may migrate into the ion-exchange sites of SAPO-34 zeolite to form active isolated Cu^2+^ species during the hydrothermal aging process, thereby causing an increase of NH_3_-SCR activity for Cu-SAPO-34 after hydrothermal aging. This may be one of the reasons for the enhanced activity of 2Cu/MCM-22 in NH_3_-SCR after hydrothermal aging. For 4Cu/MCM-22, the NO*_x_* conversion slightly decreases in low temperatures (100–200 °C) and increases in high temperatures (300–500 °C) after hydrothermal aging, as shown in Figure 9B. Peden and co-workers [78] had also observed similar results on Cu/Beta zeolites in NH_3_-SCR. They attributed the increased NO*_x_* conversion at high temperatures for Cu/Beta after hydrothermal aging to the transformation of CuO*_x_* species into CuAlO*_x_* species, as CuAlO*_x_* species have higher activity than CuO*_x_* species at high temperatures in NH_3_-SCR. As for the decreased NO*_x_* conversions in NH_3_-SCR at low temperatures after hydrothermal aging, they found that some of isolated Cu^2+^ species in Cu/Beta which were highly active transformed into less active CuO*_x_* species or CuAlO*_x_* species during the hydrothermal aging process. Peden’s observations may explain the change in NO*_x_* conversion in Figure 9B for 4Cu/MCM-22 before and after hydrothermal aging.

As shown in Figure 9C–E, when Cu loadings exceed 4 wt%, hydrothermal aging treatment has almost no influence on the performance of *x*Cu/MCM-22 zeolites, which indicates that high Cu loadings are beneficial for the hydrothermal stability of *x*Cu/MCM-22 zeolites. As discussed above, aggregated CuO*_x_* species formed in high Cu loadings caused severe blockage of the pore system in *x*Cu/MCM-22, which may have hindered the diffusion of H_2_O molecules in zeolites during the hydrothermal aging process and prevented the attack of H_2_O on the zeolite framework, and ultimately improved the hydrothermal stability of *x*Cu/MCM-22 zeolites [38,49]. The detailed mechanism behind for the hydrothermal stability of *x*Cu/MCM-22 zeolites in Figure 9 is still ambiguous now, which is currently under study. In general, *x*Cu/MCM-22 zeolites have high hydrothermal stability in NH_3_-SCR. In addition, the hydrothermal stability of *x*Cu/MCM-22 in NH_3_-SCR is related to Cu loading: when Cu loading ≤ 4 wt%, hydrothermal aging treatment slightly affects the NH_3_-SCR activity of *x*Cu/MCM-22 zeolites; once Cu loading exceeds 4 wt%, hydrothermal aging treatment has little effect on the performance of *x*Cu/MCM-22 zeolites in NH_3_-SCR.

## 4. Conclusions

A series of *x*Cu/MCM-22 zeolites with different Cu loadings were prepared by incipient wetness impregnation method and their activity and hydrothermal stability in NH_3_-SCR were investigated. SEM and XRD experiments demonstrated that Cu loading has no significant influence on the surface morphology and crystallinity of *x*Cu/MCM-22 zeolites. However, nitrogen physisorption experiments illustrated that the pore surface areas and pore volumes of *x*Cu/MCM-22 gradually decrease with Cu loadings due to the formation of aggravated CuO*_x_* nanoparticles, which cause the blockage of pore system in *x*Cu/MCM-22, especially when Cu loadings exceed 4 wt%. NH_3_-TPD experiments showed that the total acid densities of all the *x*Cu/MCM-22 zeolites are similar, and the global acid strength of *x*Cu/MCM-22 increases with Cu loadings. UV-vis, H_2_-TPR and XPS results demonstrated that three different types of Cu species exist in *x*Cu/MCM-22 zeolites: isolated Cu^2+^ species, aggregated CuO*_x_* species and unreducible copper species. In addition, the concentrations of both isolated Cu^2+^ and CuO*_x_* species in *x*Cu/MCM-22 increase with Cu loadings, but the increment of CuO*_x_* species is more distinct, especially in high Cu loadings (> 4 wt%).

Catalytic performance experiments illustrated that the NH_3_-SCR activity of *x*Cu/MCM-22 can only be affected at low Cu loadings (≤ 4 wt%); when Cu loading exceeds 4 wt%, the NO*_x_* conversions of *x*Cu/MCM-22 in NH_3_-SCR have little association with Cu loadings, due to the pore blockage effects caused by aggregated CuO*_x_* species. In general, 4Cu/MCM-22 showed the best NH_3_-SCR activity among the five *x*Cu/MCM-22 zeolites, which exhibited higher than 80% NO*_x_* conversions in a wide temperature window (160–430 °C), owing to its medium Cu loading which can not only ensure enough active isolated Cu^2+^ species but also suppress the formation of aggregated CuO*_x_* species.

The hydrothermal stability of *x*Cu/MCM-22 zeolite in NH_3_-SCR is related to its Cu loading. After hydrothermal aging in air containing 10% water at 750 °C for 10 h, the NO*_x_* conversions of 2Cu/MCM-22 in NH_3_-SCR at low temperatures (100–200 °C) obviously increased. As for 4Cu/MCM-22, the NO*_x_* conversions at low temperatures (100–200 °C) slightly decreased, but at high temperatures (300–500 °C) increased after hydrothermal aging. When Cu loading exceeds 4 wt%, hydrothermal aging treatment has almost no influence on the performances of *x*Cu/MCM-22 zeolites. In general, all of the five *x*Cu/MCM-22 zeolites with different Cu loadings showed high hydrothermal stability in NH_3_-SCR. The detailed mechanism for the high hydrothermal stability of *x*Cu/MCM-22 zeolites in NH_3_-SCR is currently under investigation.

## Figures and Tables

**Table 1:** Physicochemical properties of *x*Cu/MCM-22 zeolites with different copper contents.

Zeolites	Cu *^a^* (wt%)	RC *^b^* (%)	Surface Area *^c^* (m^2^ g^−1^)			Pore Volume *^c^* (cm^3^ g^−1^)		
			S*_BET_*	S*_micro_*	S*_ext_*	V*_total_*	V*_micro_*	V*_meso_*
H-MCM-22	0	100	500	348	152	0.50	0.163	0.337
2Cu/MCM-22	2.1	93	404	281	123	0.40	0.133	0.267
4Cu/MCM-22	3.7	100	404	282	122	0.40	0.132	0.268
6Cu/MCM-22	6.7	100	391	273	118	0.39	0.128	0.262
8Cu/MCM-22	8.5	93	365	253	112	0.38	0.118	0.262
10Cu/MCM-22	10.4	102	290	188	102	0.34	0.085	0.255

*^a^* Cu contents of all the zeolites were measured by ICP-AES. *^b^* Relative crystallinity (RC) of *x*Cu/MCM-22 was estimated by comparing the sum of the peak intensities of each zeolite at 2θ of 14.3°, 22.7°, 23.7° and 26.0° with that of H-MCM-22 as a reference, according to Xing and co-workers [40]. *^c^* Surface area and pore volume of each of the zeolites were determined by nitrogen physisorption experiments. S*_BET_*, S*_micro_* and S*_ext_* represent the BET surface area, micropore surface area and external surface area, respectively. V*_total_*, V*_micro_* and V*_meso_* represent the total pore volume, micropore volume and mesopore volume, respectively.

**Table 2:** Quantitative results of acid densities in H-MCM-22 and *x*Cu/MCM-22 zeolites.

Zeolites	Acid Sites Density (μmol/g)			
	Total *^a^*	Weak *^b^*	Medium *^b^*	Strong *^b^*
H-MCM-22	1062	255	446	361
2Cu/MCM-22	1216	207	328	681
4Cu/MCM-22	1124	202	247	674
6Cu/MCM-22	1152	173	265	714
8Cu/MCM-22	1197	96	323	778
10Cu/MCM-22	1276	102	140	1034

*^a^* Total acid density of each sample was determined by NH_3_-TPD experiments in Figure 3. *^b^* Densities of weak, medium and strong acid sites were calculated based on the deconvoluted relative percentages of the peaks at around 140, 210 and 460 °C in Figure 3 and Figure S3, respectively.

**Table 3:** Quantitative results of H_2_-TPR experiments in *x*Cu/MCM-22 zeolites.

Catalyst	Reduction Process 1*^a^* (Cu^2+^ to Cu^+^)		Reduction Process 2*^a^* (CuO to Cu^0^)		Reduction Process 3*^a^* (Cu^+^ to Cu^0^)		H_2_ Consumpt-ion^*b*^ (µmol/g)	H_2_/Cu *^c^*
	Peak Temperature (°C)	Relative Peak Area (%)	Peak Temper-ature (°C)	Relative Peak Area (%)	Peak Temper-ature (°C)	Relative Peak Area (%)		
2Cu/MCM-22	196	21	256	14	386 580	65	241	0.73
4Cu/MCM-22	170	23	235	19	417 580	58	428	0.74
6Cu/MCM-22	177	34	235	31	352	35	622	0.59
8Cu/MCM-22	170	27	235 260	48	300	25	792	0.59
10Cu/MCM-22	161	29	217 242	58	338	13	976	0.60

*^a^* Relative quantitative results of each Cu/MCM-22 zeolite according to the deconvoluted peaks shown in Figure S4. Reduction processes 1, 2 and 3 represent the reductions of Cu^2+^ to Cu^+^, CuO to Cu^0^ and Cu^+^ to Cu^0^, respectively. *^b^* Total H_2_ consumption of each sample was recorded by a thermal conductivity detector (TCD). *^c^* H_2_/Cu ratio was calculated from the total H_2_ consumption value divided by the Cu concentration in each Cu/MCM-22 zeolite (Table 1).

**Table 4:** XPS and ICP-AES results of *x*Cu/MCM-22 zeolites.

Zeolites	Si/Al *^a^* mol/mol	Si/Cu *^a^* mol/mol	Si/Al *^b^* mol/mol	Si/Cu *^b^* mol/mol
2Cu/MCM-22	10.3	121.8	11.0	33.5
4Cu/MCM-22	5.9	53.2		19.6
6Cu/MCM-22	6.8	50.6		7.4
8Cu/MCM-22	7.7	46.3		11.3
10Cu/MCM-22	6.3	41.4		7.2

*^a^* Si/Al and Si/Cu ratios were obtained from XPS. *^b^* Si/Al and Si/Cu ratios were obtained from ICP-AES.

## Supplementary Materials

The following are available online at https://www.mdpi.com/2079-4991/10/11/2170/s1. Figure S1: Relationship between the intensity of CuO diffraction peaks and Cu contents over xCu/MCM-22 zeolites. Figure S2: Correlations between the loss percentages of (A) surface areas (SBET, BET surface area, and Smicro, micropore surface area) and (B) pore volumes (Vtotal, total pore volume, and Vmicro, micropore volume) with Cu contents over xCu/MCM-22 zeolites. Figure S3: Deconvolution of the NH3-TPD profiles of H-MCM-22 and xCu/MCM-22 zeolites. Figure S4: Deconvolution of the H2-TPR profiles of xCu/MCM-22 with different copper contents. Figure S5: H2-TPR profile of 6Cu/MCM-22.
