# Supplementary material for: Catalytic Performances of Cu/MCM-22 Zeolites with Different Cu Loadings in NH3-SCR
Source: Nanomaterials (Basel). 2020 Oct 30;10(11):2170. doi: 10.3390/nano10112170 (PMC7694057; doi:10.3390/nano10112170)
Supplement: Supplementary file 1 [file nanomaterials-10-02170-s001.pdf]

nanomaterials

Supporting Information

Catalytic Performances of Cu/MCM-22 Zeolites with Different Cu Loadings in NH_3-SCR

Jialing Chen [1,*], Gang Peng [1], Tingyu Liang [2], Wenbo Zhang [1], Wei Zheng [1], Haoran Zhao [1], Li Guo [1,*] and Xiaoqin Wu [1,*]

[1] Key Laboratory of Hubei Province for Coal Conversion and New Carbon Materials, School of Chemistry and Chemical Engineering, Wuhan University of Science and Technology, Wuhan 430081, China; penggang@btrchina.com (G.P.); wustzhangwenbo@163.com (W.Z.); zhengwei321@126.com (W.Z.); Zhaohr290370@163.com (H.Z.)

[2] Key Laboratory for Green Chemical Process of Ministry of Education, and Hubei Key Laboratory of Novel Reactor & Green Chemical Technology, School of Chemical Engineering & Pharmacy, Wuhan Institute of Technology, Wuhan 430205, China; ltingyu2006@yahoo.com

* Correspondence: chenjialing@wust.edu.cn (J.C.); guoli@wust.edu.cn (L.G.); wuxiaoqin@wust.edu.cn (X.W.)

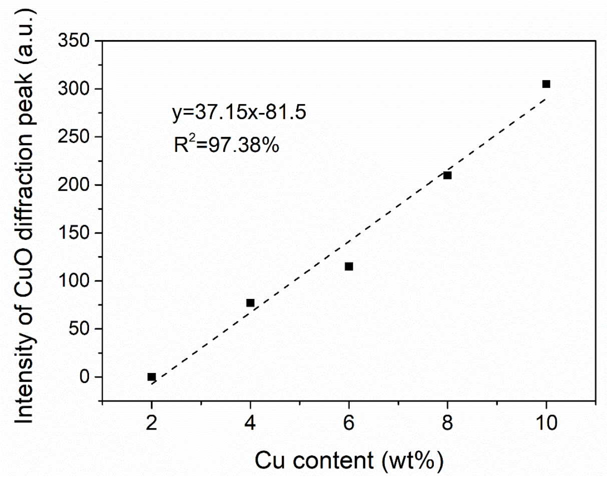

Figure S1. Relationship between the intensity of CuO diffraction peaks and Cu contents over *x*Cu/MCM-22 zeolites.

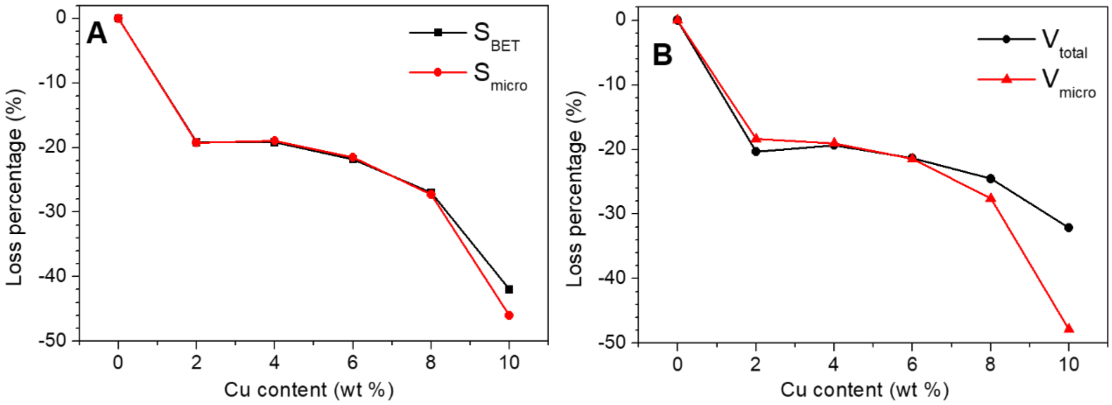

Figure S2. Correlations between the loss percentage of (A) surface areas (S_{BET}, BET surface area, and S_{micro}, micropore surface area), or (B) pore volumes (V_{total}, total pore volume, and V_{micro}, micropore volume) with Cu contents over *x*Cu/MCM-22 zeolites.

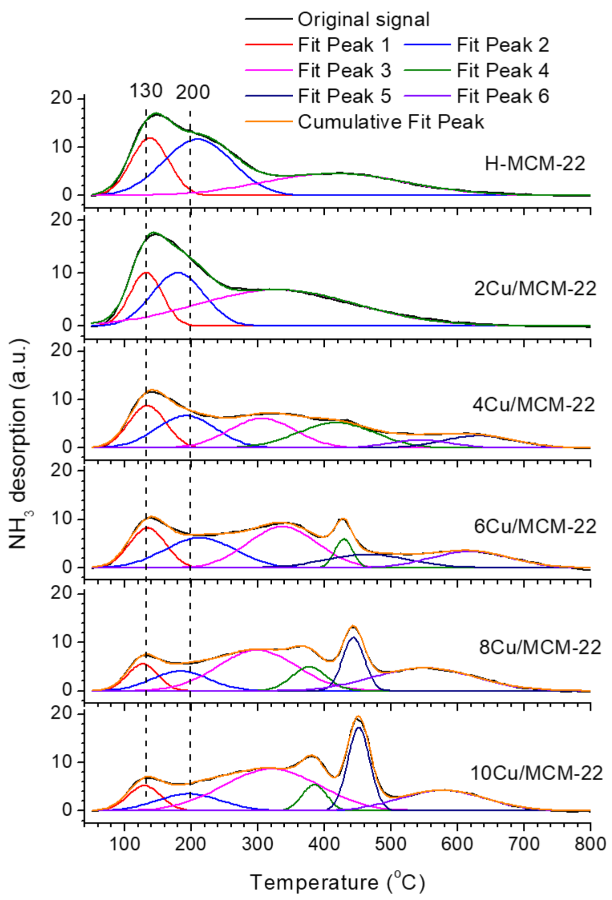

Figure S3. Deconvolution of the NH_3-TPD profiles of H-MCM-22 and *x*Cu/MCM-22 zeolites.

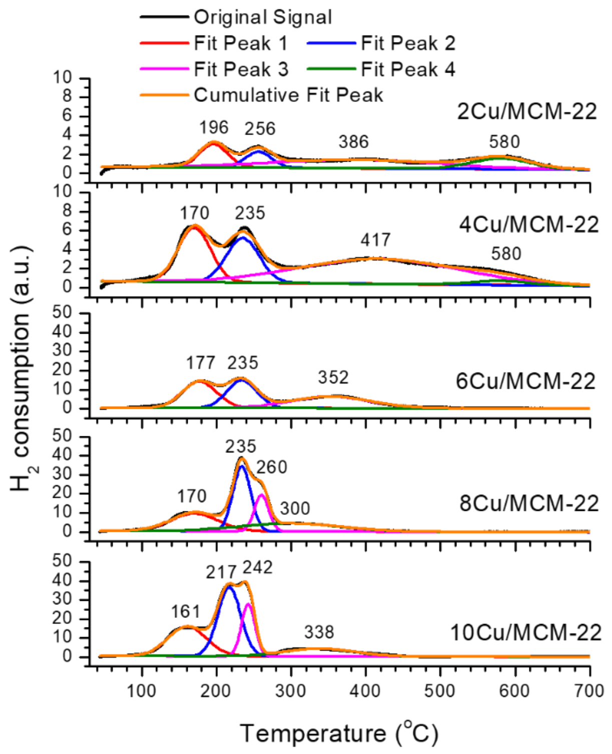

Figure S4. Deconvolution of the H_2-TPR profiles of *x*Cu/MCM-22 with different copper contents.

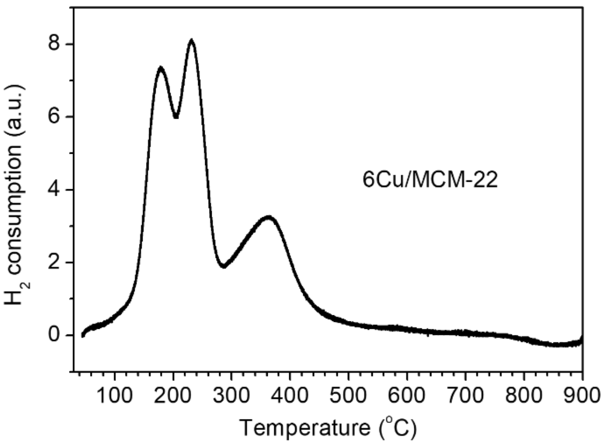

Figure S5. H_2-TPR profile of 6Cu/MCM-22.
